# Chemo-enzymatic synthesis and *in vitro* cytokine profiling of tailor-made oligofructosides

**DOI:** 10.1186/1472-6750-12-90

**Published:** 2012-11-26

**Authors:** Arne Homann, Malte Timm, Jürgen Seibel

**Affiliations:** 1 Department of Organic Chemistry, University of Wuerzburg, Am Hubland, 97074, Wuerzburg, Germany; 2 Present address: Research Center Borstel, Parkallee 1-40, 23845, Borstel, Germany

**Keywords:** Oligofructoside, Glycosyltransferase, Suc1, *Aspergillus niger*, SacB, *Bacillus megaterium*, CXCL8 (IL-8), CCL2 (MCP-1), Caco-2

## Abstract

**Background:**

It is well known that carbohydrates play fundamental roles in cell signaling and infection processes as well as tumor formation and progression. However, the interaction pathways and cellular receptors targeted by carbohydrates and glycoconjugates remain poorly examined and understood. This lack of research stems, at least to a major part, from accessibility problems of large, branched oligosaccharides.

**Results:**

To test glycan - cell interactions *in vitro*, a variety of tailored oligosaccharides was synthesized chemo-enzymatically. Glycosyltransferases from the GRAS organisms *Bacillus megaterium* (SacB) and *Aspergillus niger* (Suc1) were used in this study. Substrate engineering of these glycosyltransferases generally acting on sucrose leads to the controlled formation of novel tailored di-, tri- and tetrasaccharides. Already industrially used as prebiotics in functional food, the immunogenic potential of novel oligosaccharides was characterized in this study. A differential secretion of CXCL8 and CCL2 was observed upon oligosaccharide co-cultivation with colorectal epithelial Caco-2 cells.

**Conclusion:**

Pure carbohydrates are able to stimulate a cytokine response in human endothelial cells *in vitro*. The type and amount of cytokine secretion depends on the type of co-cultivated oligosaccharide.

## Background

Inflammation processes are essential for the immune system of a host organism attacked by bacteria, viruses or other immunogenic molecules. However, persistent inflammation is a pathologic indication. The intestine, being the largest barrier of the human body to the environment, is under a state of persistent controlled inflammation because of its permanent contact with the gut microbiota. Intestinal epithelial cells release cytokines and chemokines upon external stimulation, *e.g.* by bacteria and their surface structures [1]. The factors which trigger inflammation and the release or suppression of cytokines and chemokines have been investigated thoroughly over the last decade, but the process is still not fully understood. Clearly, cytokine secretion can be triggered by lipopolysaccharide (LPS) on the surface of Gram-negative bacteria [2,3] or capsular polysaccharides and lipoteichoic acid from Gram-positive species [4,5].

Oligo- and polysaccharides containing fructose have been known for several years as prebiotics [6,7]. Fructose recently was described as a signaling molecule and lead structure for carbohydrates with enhanced antigenicity in HIV vaccination [8]. The extent of the fructan oligo- and polymerization was described as controllable in an enzymatic synthesis process [9]. Fructosyltransferases like inulosucrases and levansucrases which synthesize fructans of various chain lengths are common in many different bacteria including the gut microbiota [10]. The challenges to access large, branched oligosaccharides using chemical synthesis, may be overcome using chemo-enzymatic approaches [11-13]. Sucrose analogues synthesized by SacB from *B. megaterium* were used as precursors for the synthesis of oligofructosides with the fructosyltransferase Suc1 from *A. niger*[14]. For the present study, the enzymatic synthesis process was scaled up to yield biological test amounts. The tailored oligofructosides tested in this study are capped by the monosaccharides d-glucose, d-mannose, d-galactose, d-fucose or d-xylose, elongated with fructosyl units under tight control of the degree of polymerization. These oligofructosides are supposed to mimic the structural characteristics of immunogenic carbohydrate patterns of antigens, thus triggering the release of cytokines and/or chemokines.

## Results

### Chemo-enzymatic synthesis of novel oligofructosides by substrate engineering of fructosyltransferases

**Figure 1:**
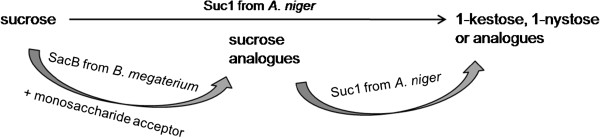
**Enzymatic synthesis of novel oligofructosides.** Oligofructosides used in this study were synthesized by the concerted action of two fructosyltransferases from *B. megaterium* (SacB) and *A. niger* (Suc1).

**Figure 2:**
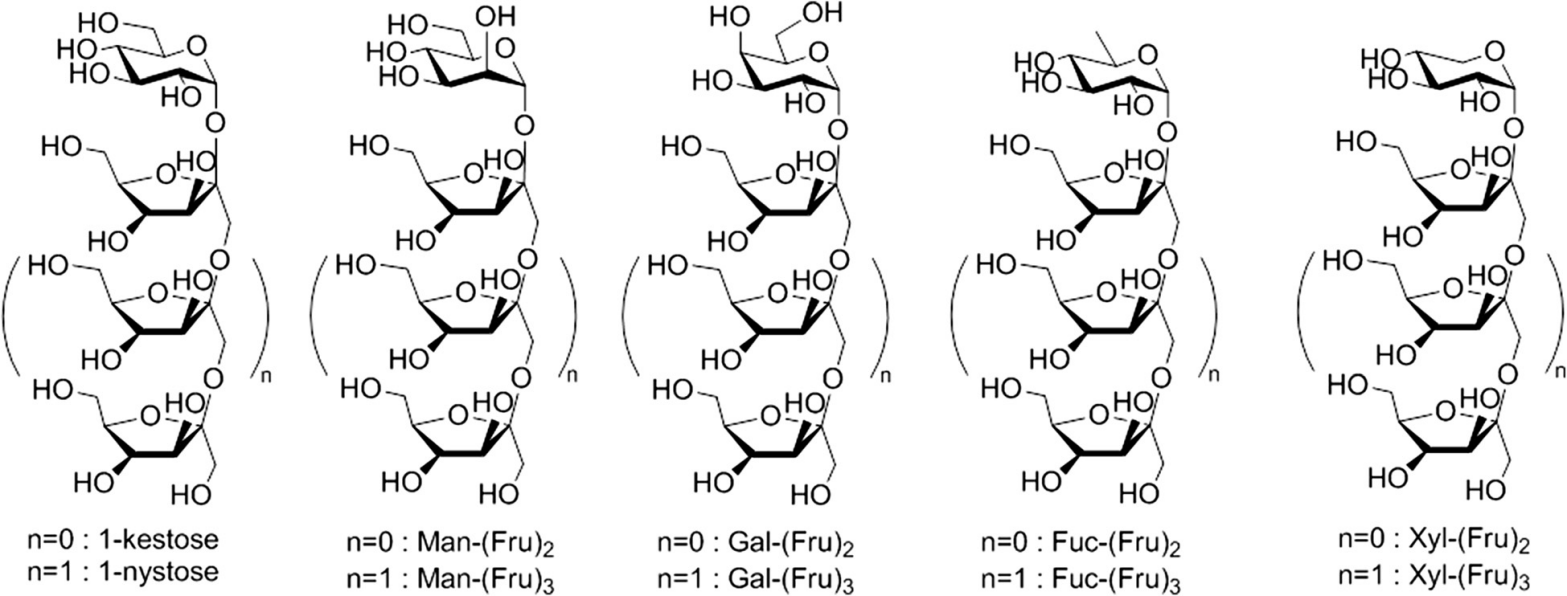
**Structure of tailored oligofructosides tested in terms of their immunological properties.** Controlled enzymatic synthesis was based on sucrose analogue precursors and the addition of a variable fructosyl backbone.

Tailored oligofructosides were synthesized, purified and characterized regarding their composition and stereochemistry. Substrate engineering of two fructosyltransferases from GRAS organisms (SacB from *B. megaterium* and Suc1 from *A. niger*) leads to novel tailor-made oligofructosides of defined fructosyl backbone lengths (Figures 1 and 2). The first step in their enzymatic synthesis is the formation of the α-(1,2) linked disaccharide (sucrose analogue). The fructosyltransferase SacB from *B. megaterium* provides access to the efficient synthesis of sucrose analogues (Gal-Fru, Man-Fru, Xyl-Fru and Fuc-Fru) under appropriate reaction conditions. The synthesis reaction was performed according to the process of sucrose analogue synthesis by SacB from *B. subtilis*[15]. However, SacB from *B. megaterium* proved to be much more efficient in terms of chemo-enzymatic synthesis with an increased substrate affinity (K_m_ 6.6 compared to 14) and turnover number (k_cat_ 2200 as opposed to 165) [16].

**Figure 3:**
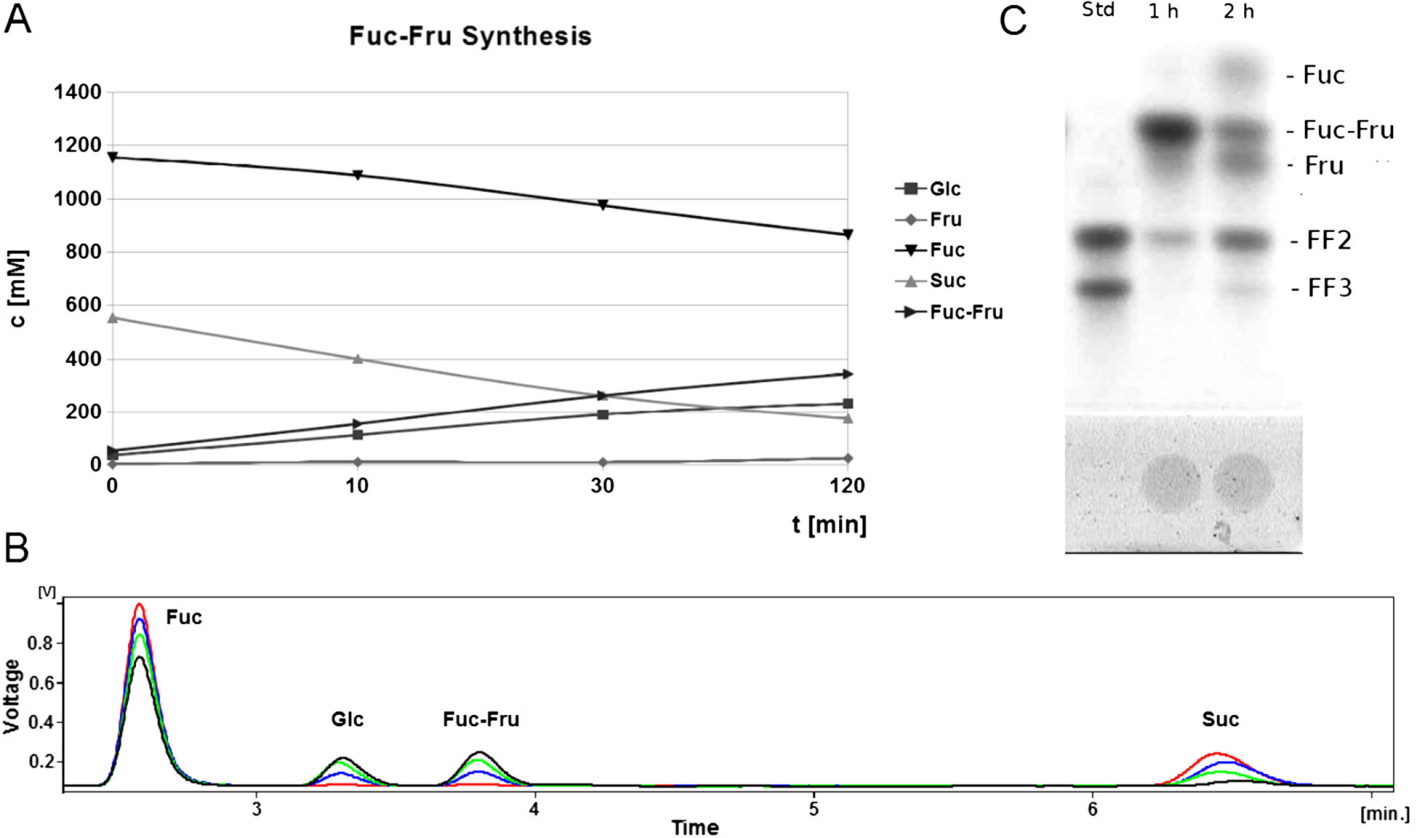
**Synthesis of the oligofructosides capped by fucose.A**, the acceptor reaction for the fucosyl-containing disaccharide Fuc-Fru was analyzed by HPAEC. Reaction conditions were: Fucose (1.2 M), sucrose (600 mM) in phosphate buffer after Sörensen (50 mM, pH 6.6), 10 mg l^-1^ fructosyltransferase SacB, at 200 rpm and 37°C for 2 h. **B**, the same acceptor reaction as in A shown as HPAEC chromatogram. Carbohydrates corresponding to the peaks are indicated. Analysis time points are indicated as follows. Red, 0 min, blue, 10min, green, 30 min and black, 2 h reaction time. **C**, The transfructosylation reaction yielding the fucosyl-capped tri- and tetrasaccharides Fuc-(Fru)_2_ and Fuc-(Fru)_3_ was performed using Suc1-containing culture supernatant 1:50 (v/v), 500 mM Fuc-Fru in Sörensen`s phosphate buffer (50 mM, pH 5.6), at 45°C and 200 rpm.

Each chemo-enzymatic synthesis process was analyzed by HPAEC. As an example, the process for Fuc-Fru synthesis is shown in Figure 3A. The distinct addition of the second and third unit of the fructosyl backbone was performed by the fructosyltransferase Suc1 from *A. niger*. Suc1 is highly specific for the synthesis of defined tri- and tetrasaccharides, 1-kestose (Glc-Fru_2_) and 1-nystose (Glc-Fru_3_) and their analogues depending on the reaction conditions. Trisaccharide kestose analogues are Gal-(Fru)_2_, Man-(Fru)_2_, Xyl-(Fru)_2_, and Fuc-(Fru)_2_. Tetrasaccharide nystose analogues containing three fructosyl moieties are Gal-(Fru)_3_, Man-(Fru)_3_, Xyl-(Fru)_3_ and Fuc-(Fru)_3_ (Figure 2). The previously described products [14] as well as the novel fucosyl-capped tri- and tetrasaccharides were identified and analyzed by TLC and HPAEC. Scale-up of the synthesis process yields pure oligofructosides in cell culture test amounts (mg-scale, Table 1). As an example, the synthesis process for Fuc-Fru_2_ and Fuc-Fru_3_ is shown in Figure 3B.

**Table 1:** **Reaction times and yields for the oligosaccharide synthesis by the fructosyltransferase Suc1 from *A. niger***

	**t**	**Conversion**
	**[min]**	**[% mol mol**^**-1**^**]**
1-kestose	18	81
1-nystose	60	93
MF_2_	60	71
MF_3_	180	87
GF_2_	420	44
GF_3_	960	65
XF_2_	20	75
XF_3_	120	94
FF_2_	60	65
FF_3_	120	88

Reaction conditions were Suc1 enzyme dilution 1:50 (v/v), 500 mM sucrose analogue to be converted in Sörensen`s phosphate buffer (50 mM, pH 5.6) at 45°C and 200 rpm. The reaction time depending on the oligosaccharide to be synthesized is indicated.

### Oligofructoside-stimulated Caco-2 cells differentially secrete CXCL8 and CCL2

**Figure 4:**
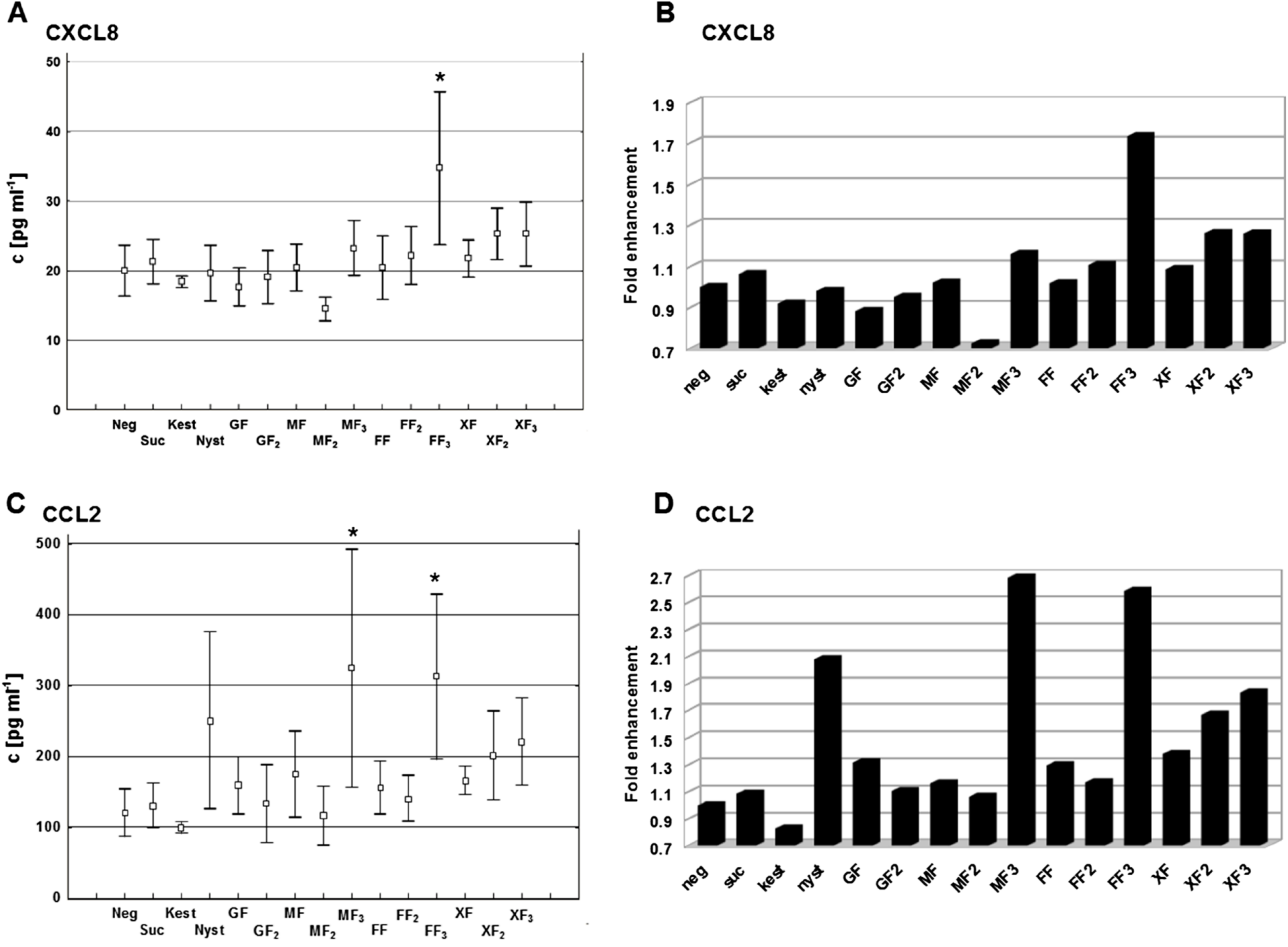
**CXCL8 and CCL2 secretion level upon oligofructoside co-incubation of Caco-2 cells.A**, **C** Secretion level of CXCL-8 (**A**) and CCL2 (**C**) after co-incubation with the oligofructoside indicated. **B**, **D** Means of **A**, **C** were calculated as enhancement factors relative to cytokine/chemokine secretion level of the negative control. Significant differences according to the Fischer algorithm are indicated by a star. In A, FF_3_ is significantly different from all other oligofructosides except for XF_2_ and XF_3_. In C, FF_3_ and MF_3_ differ significantly from MF_2_ and kest. Caco-2 cells were incubated in the presence of 25 μM oligofructoside at 37°C, 5% CO_2_ for 48 h.

The novel synthesized oligofructosides were tested in terms of immunostimulating properties on human epithelial Caco-2 cells. This cell line is a model for the absorption of pharmacological products in the intestinal region [17,18]. 25 cytokines and chemokines were analyzed in the growth medium during co-cultivation with the novel oligofructosides (Eotaxin, GM-CSF, IFN-α, IFN-γ, IL-1RA, IL- 1β, IL-2, IL-2R, IL-4, IL-5, IL-6, IL-7, IL-8, IL-10, IL-12p40/p70, IL-13, IL-15, IL-17, IP-10, MCP-1, MIG, MIP-1α, MIP-1β, RANTES and TNF-α, 25-plex cytokine analysis kit, Biosource, Invitrogen). Caco-2 cells were grown for 48 h in 24-well dishes containing the oligofructosides to be investigated in a concentration of 25 μM. Five-fold repeats of the experiment for each carbohydrate (four-fold for MF_2_) ensured the reproducibility of the results. CXCL8 (also known as interleukin 8, IL-8) and CCL2 (monocyte chemoattractant protein, MCP-1) were the only cytokines/chemokines in the media supernatants which led to a significant signal detected by the luminex system. Both standard curve fits show a correlation of over 99% (data not shown). The measured fluorescence intensities are in the linear part of the standard curve indicating the reliability of the data. The other 23 cytokines and chemokines analyzed showed no significant response under these conditions. CCL2 generally shows a higher signal-to-background enhancement compared to the cultivation of Caco-2 cells without added carbohydrates. The CCL2 release is increased up to a concentration of 320 pg ml^-1^ (Man-Fru_3_, Figure 4C, D). The characteristics of a longer fructosyl backbone seem to enhance this effect. For the tetrasaccharides 1-nystose (250 pg ml^-1^), Man-Fru_3_ (320 pg ml^-1^), Fuc-Fru_3_ (310 pg ml^-1^) and Xyl-Fru_3_ (220 pg ml^-1^), the release of CCL2 is clearly triggered (Figure 4C, D). The stimulation of CXCL8 is also observable when the fucose-containing 1-nystose analogue Fuc-Fru_3_ is added (35 pg ml^-1^). Also in the case of CXCL8, longer fructosyl chains seem to enhance the release of this cytokine. In the samples containing Man-Fru_3_ (24 pg ml^-1^), Fuc-Fru_3_ (35 pg ml^-1^) and Xyl-Fru_3_ (25 pg ml^-1^) the highest concentrations of CXCL8 were detectable (Figure 4A, B). CXCL8 and CCL2 release was consistently not enhanced when incubated with the trisaccharides 1-kestose and Man-Fru_2_ (Figure 4).

## Discussion

Co-incubation of human epithelial Caco-2 cells with certain types of pure, unconjugated oligofructosides leads to enhanced secretion of CXCL8 and CCL2. CXCL8 is a potent inflammation marker recruiting neutrophils to sites of infection. It is secreted by various cell types including epithelial cells [19]. CCL2 is described as an effective chemoattractor for monocytes from the blood stream [20]. Our results show that CCL2 release can be clearly triggered by the tetrasaccharide 1-nystose and even more enhanced by the nystose analogues Man-Fru_3_ and Fuc-Fru_3_. The observed differential cytokine secretion pattern raises questions: Is this stimulating effect dependent on the carbohydrate structure and if so, which structural elements trigger or suppress the release of cytokines and chemokines? Because of the differential secretion pattern and only two significant signals (out of 25 investigated), it was shown that cytokine secretion by Caco-2 cells in this assay is dependent on the oligofructoside type. But why are just 2 significant signals of secreted proteins detectable? One possibility is, that epithelial cells in the intestine only have a restricted repertoire of cytokines to be synthesized and secreted. Another point is, that this cell type is in constant contact with ubiquitous nutrients and commensal gut bacteria. Hence, it may have evolved tolerance against certain alien structures. The intestine being the largest barrier of the human body to the environment, has a special set of immunologically active cells. This area is under a state of persistent controlled inflammation because of its permanent contact with the gut microbiota. Special intestinal macrophages (IMACs) mediate tolerance to beneficial gut bacteria. Perturbations of these processes, like the release of CCL2, inhibit the differentiation of macrophages to IMACs thus leading to (chronic) inflammatory bowel diseases (IBDs) [21]. Intestinal epithelial cells release cytokines and chemokines upon external stimulation, *e.g.* by bacteria and their surface structures [1]. The factors which trigger inflammation and the release or suppression of cytokines and chemokines have been investigated thoroughly over the last decade, but the process is still not fully understood.

In this study, mannose- and fucose-capped oligofructosides generally evoke the highest increase in CCL2 and CXCL8 release (Figure 4). This might be due to their participation in natural cell-cell communication processes. Fucose often is a branching carbohydrate unit *e.g.* in the Lewis X motif. This motif is known as immunogenic under certain conditions, e.g. incomplete sialylation. Mannose is part of the core N-glycan structure. Its exposition often leads to the release of cytokines, *e.g.* CCL2 in mannosidase knock-out mice [22]. Interestingly, the different monosaccharide cap structure of the fructosyl backbone is not the only factor influencing the release of CXCL8 and CCL2, but also the length of the fructosyl backbone. For example, CCL2 secretion is triggered by 1-nystose and its tetrasaccharide analogues Man-Fru_3_ and Fuc-Fru_3_ but suppressed by kestose and its analogue Man-Fru_2_ (Figure 4). Thus, stereochemical and spatial aspects of oligosaccharides obviously have to be considered in terms of cell signalling processes. Recently, it was described that the different shape of bacterial lipopolysaccharide (LPS) determines which receptor is targeted and thus how cell signalling is processed [23-25]. The potential target receptors which are known to act competitively are shown in Additional file 1: Figure S1. The differential secretion of cytokines and thus the induction of an inflammatory response by the interaction of these receptors is still a scientific area with many long-standing questions.

## Conclusions

Carbohydrates are ubiquitious structures on the surface of a plethora of different cell types including potentially pathogenic and beneficial gut bacteria. Auto-immune diseases like Crohn`s disease are linked to persistent, pathologic inflammation. As abundant surface structures of host and pathogen cells, carbohydrates may play an important role in the induction of inflammation and tolerance, respectively. Advances in carbohydrate research in combination with cell biology and immunology methods may lead to a detailed understanding of inflammation processes. The pure, tailored carbohydrate structures examined in this study induce such a differential secretion of cytokines in endothelial cells *in vitro*. Further advances in oligosaccharide synthesis lead broadened possibilities to investigate *in vivo* inflammation mechanisms of carbohydrate-cell receptor crosstalk. Controlled stimulation of the immune system may be one component towards a successful treatment of auto-immune diseases.

## Methods

### Chemo-enzymatic synthesis of tailored oligofructosides

The fructosyltransferases from the GRAS organisms *B. megaterium* (SacB) and *A. niger* (Suc1) were used for the synthesis of a fructosyl-based carbohydrate backbone capped with different types of monosaccharides (glucose, galactose, mannose, fucose and xylose).The oligofructosides were synthesized in two steps. First, sucrose analogues were synthesized by the fructosyltransferase SacB from *B. megaterium*. After analysis and purification, the elongation reaction was performed by the fructosyltransferase Suc1 from *A. niger*.

### Synthesis and purification of sucrose analogues by the fructosyltransferase SacB from *bacillus megaterium*

For the synthesis of sucrose analogues, the acceptor monosaccharide was used in a concentration of 1.2 M. The transfructosylation reaction was performed with added sucrose (600 mM) in phosphate buffer after Sörensen (50 mM, pH 6.6). SacB was applied in a final concentration of 10 mg l^-1^ at 200 rpm and 37°C for 2 h in a 1.5 ml or 15 ml reaction tube. The resulting sucrose analogues were analyzed qualitatively and quantitatively by thin-layer chromatography (TLC, 2.2) and high-performance anion exchange chromatography (HPAEC, 2.3). The purification of the sucrose analogues was performed by a silica column with a carbohydrate-containing mobile phase (60% ethylacetate, 30% isopropanol, 10% water, all v/v). The products were analyzed by TLC and HPAEC.

### Synthesis and purification of 1-kestose, 1-nystose and analogues by the fructosyltransferase Suc1 from *aspergillus Niger*

The subsequent synthesis step of 1-kestose, 1-nystose and their analogues was performed by the fructosyltransferase Suc1 from *A. niger* as described previously with the sucrose analogues synthesized by SacB from B. subtilis [14]. Briefly, the supernatant of a cultivation of *A. niger* SKAN1015 was used in a dilution of 1:50 (v/v). The Suc1 dilution was mixed with 500 mM of the sucrose analogue to be converted in Sörensen`s phosphate buffer (50 mM, pH 5.6). The reaction was performed at 45°C and 200 rpm. The reaction time depends on the desired oligofructoside to be synthesized [14].The purification of 1-kestose, 1-nystose and their analogues was performed by size exclusion chromatography. An open chromatography gel filtration system was used (Biogel, Bio-Rad) and degassed water containing the carbohydrates to be separated as mobile phase.

### Analysis of carbohydrates by thin-layer chromatography (TLC)

The sample was diluted to a total carbohydrate concentration of 1–3 g l^-1^. 3 μl of the sample was applied on a TLC plate (TLC aluminium foil coated with silica 60, 20 x 20 cm with concentration zone, Merck). After drying the TLC was run in a TLC chamber equilibrated with the mobile phase. After 45 min the plate was dried and again incubated for 45 min. The staining of the carbohydrates was performed by a short dive into the developing solution (sulfuric acid 5% (v/v) N-(1-naphtyl) ethylendiamine dihydrochloride 0.3% (w/v) in methanol) and incubation at 150°C for 5 min. An appropriate standard has to be applied each time (here: glucose 0.1 g l^-1^, fructose 0.1 g l^-1^, sucrose 0.1 g l^-1^, 1-kestose 0.1 g l^-1^, 1-nystose 0.1 g l^-1^).

### Analysis of carbohydrates by high-performance anion exchange chromatography (HPAEC)

HPAEC analysis was used to determine the kinetic parameters of the enzyme reactions and the optimal reaction conditions. The HPAEC is a modular high-performance liquid chromatography optimized for the analysis of carbohydrates. The pre-column (CarboPac PA1, 4*50 mm, Dionex) and the following seperation column (CarboPac PA1 4*250 mm, Dionex)) of the HPAEC device are used to separate the carbohydrates with a gradient of the eluent (1 M sodium acetate in 0.1 M sodium hydroxide in MilliQ, Millipore, deionized water, protocol see Table 2). The samples were applied by an autosampler (Perkin Elmer). A degaser unit was used for removing oxygen and carbon dioxide from the mobile phase (sodium hydroxide, 100 mM in water) and the eluent (sodium hydroxide, 100 mM and sodium acetate, 1 M in water). A thermostat ensured a stable temperature of 15°C. The flow rate was 1 ml min^-1^. The total carbohydrate concentration has to be set to 100 – 200 mg l^-1^ correlating with the used detector sensitivity of “1k”. The chromatograms were recorded with the software Clarity (Ver. 2.4.1.77, DataApex).

**Table 2:** HPAEC eluent gradient program

	
0 - 5 min	0% 1 M NaAc
5 - 25 min	to 25% 1 M NaAc
25 - 30 min	to 50% 1 M NaAc
30 - 35 min	50% 1 M NaAc
35 - 37 min	to 0% 1 M NaAc
37 - 60 min	0% 1 M NaAc

### Co-cultivation of Caco-2 cells with tailor-made oligofructosides

Caco-2 cells were cultivated in Dulbecco`s modified Eagle`s medium (DMEM)/HamsF12 (Gibco) supplied with 10% fetal calf serum (FCS) and 200 μg l^-1^ ampicillin at 37°C and 5% CO_2_. At 80% confluency, cells were split in a ratio of 1:10. For the oligofructoside assay, Caco-2 cells at 80% confluence were cultivated in 24-well dishes (Gibco). The split ratio was 1:10 and each well was supplied with the carbohydrate to be tested in a concentration of 25 μM. After 48 h, from each well a sample of the media supernatant was collected for cytokine analysis.

### Cytokine and chemokine detection assay

For the oligofructoside assay, Caco-2 cells at 80% confluence were split as described and cultivated in 24-well dishes (Biochrom). Each well was supplied with the oligofructoside to be tested (final concentration 25 μM). After 48 h (80% confluence) the supernatant medium was collected for cytokine analysis. The assay was performed with a 25-plex human cytokine analysis kit according to the manufacturer`s instructions (Biosource, Invitrogen). Briefly, the supernatant medium was incubated with antibody-functionalized beads and detected with biotinylated secondary antibodies. Streptavidin-R-phycoerythrin was used as fluorescence marker. The final analysis was performed by the luminex system which recognizes spectral properties of the beads and quantifies the bead load by the specific fluorescence intensity. 25 cytokines were analyzed in parallel per sample (Eotaxin, GM-CSF, IFN-α, IFN-γ, IL-1RA, IL- 1β, IL-2, IL-2R, IL-4, IL-5, IL-6, IL-7, IL-8, IL-10, IL-12p40/p70, IL-13, IL-15, IL-17, IP-10, MCP-1, MIG, MIP-1α, MIP-1β, RANTES, TNF-α) according to the manufacturer`s instructions (luminex system, Qiagen).

## Abbreviations

Gal-Fru: GF, α-d-galactopyranosyl-(1,2)-β-d-fructofuranoside; Man-Fru: MF, α-d-mannopyranosyl-(1,2)-β-d-fructofuranoside; Xyl-Fru: XF, α-d-xylopyranosyl-(1,2)-β-d-fructofuranoside; Fuc-Fru: FF, α-d-fucopyranosyl-(1,2)-β-d-fructofuranoside.

## Competing interests

The authors declare no competing interests.

## Authors’ contributions

AH and MT performed experiments. AH and JS designed the experiments and analysed the data. AH and JS wrote the manuscript. All authors read and approved the manuscript.

## Supplementary Material

Additional file 1: Figure S1 Lipopolysaccharide interactions with cell surface-located Toll-like receptors 2 and 4. The different shapes of bacterial lipopolysaccharides (LPS) are keys for the identification of their target receptor (simplified adaption from [17]). Here, the shape of LPS ligands is determined by the grade of fatty acid substitution. The distinct receptor binding mode depending on the molecular conformation is supposed to be mimicked by the oligosaccharides tested in this study. Click here for file
