# Supplementary material for: Chemo-enzymatic synthesis and in vitro cytokine profiling of tailor-made oligofructosides
Source: BMC Biotechnol. 2012 Nov 26;12:90. doi: 10.1186/1472-6750-12-90 (PMC3576278; doi:10.1186/1472-6750-12-90)

**Supporting Information**

**Supporting Methods**

**Analysis of carbohydrates by nuclear magnetic resonance (NMR)**

All samples to be analysed by NMR were freeze-dried and dissolved in D_2_O. Spectra were recorded on a Bruker DRX600 or Bruker DRX400 operating at 600 and 400 MHz, respectively. One-dimensional ^1^H and ^13^C spectra and phase-sensitive two-dimensional spectra (double-quantum-filtered COSY, NOESY (Nuclear overhauser and exchange spectroscopy), TOCSY (Total correlated spectroscopy), ^1^H-^13^C HSQC (Heteronuclear single quantum coherence) and HMBC (Heteronuclear multiple bond correlation)) were recorded using standard pulse programs at 38.8 °C, and data were analysed using the software packages XWINNMR (Bruker, Germany). Chemical shifts are calculated in the δ-scale (ppm) and coupling constants *J* in Hz.

**Analysis of carbohydrates by electrospray-ionisation mass spectrometry (ESI-MS)**

Aliquots (1–3 μL) corresponding to 2–20 pmol of oligosaccharides were applied to a nanospray gold-coated glass capillary placed orthogonally in front of the entrance hole of a QTOF-II instrument (Micromass, UK). Then, 1000 V was applied to the capillary and ions were separated by the time-of-flight (TOF) analyser. For MS/MS analysis parent ions were selected by the quadrupole mass filter and subjected to collision-induced dissociation. Resulting daughter ions were then separated by the TOF-analyser.Electrospray-ionisation mass spectra (ESI-MS) were recorded with a Finnigan MAT 8340 on samples dissolved in CH_3_OH.

**Supporting Results**

**β-d-Fructofuranosyl-α-d-mannopyranoside (Man-Fru)**

*R*_f_ 0.40 (6:3:1 EtOAc-Isopropanol-H_2_O, 3 ascends); ^1^H NMR (400 MHz, D_2_O) *δ* 5.30-5.29 (d, *J* = 1.9 Hz, 1H, 1-H), 4.14-4.12 (d, *J* = 8.7 Hz, 1H, 3’-H), 4.02-3.99 (t, *J* = 8.7 Hz, 1H, 4’-H), 3.86-3.67 (m, 9 H, 2-H, 3-H, 4-H, 5-H, 6-H_2_, 5’-H, 6’-H_2_), 3.61 (s, 1’-H_2_). ^13^C NMR (100 MHz, D_2_O) *δ* 106.55 (C-2’), 96.18 (C-1), 83.93 (C-5’), 78.58 (C-3’), 76.51 (C-4’), 75.91 73.68 72.70 (C-2, C-3, C-5), 69.02 (C-4), 64.98 (C-6’), 63.55 (C-1’), 63.21 (C-6).

ESI-MS: *m/z*: calcd for C_12_H_22_O_11_Na: 365.0, found 365.0 [M+Na]^+^**.**

**β-d-Fructofuranosyl-(2→1)-*β*- d-fructofuranosyl-(2→1)-*α*- d-mannopyranoside (Man-Fru_2_)**

*R_f_* = 0.23 (acetonitrile/water 8:2, 3 ascents^1^H NMR (400 MHz, D_2_O) *δ* 5.31 (d, *J=*1.6 Hz, 1H; 1-H), 4.19-4.17 (d, J=8.6 Hz, 1H; 3’-H), 4.19-4.17 (d, J=8.3 Hz, 1H; 3’’-H), 4.06-4.03 (t, J=8.2 Hz, 1H; 4’’-H), 4.03-3.99 (t, *J=*8.5 Hz, 1H; 4’-H), 3.88-3.84 (m, 2H; 2-H, 5-H), 3.84-3.81 (m, 2H; 5’-H, 5’’-H), 3.88-3.84 (m, 12H; 3-H, 4-H, 6-H_2_, 1’-H_2_, 6’-H_2_, 1’’-H_2_, 6’’-H_2_). ^13^C NMR (100 MHz, D_2_O): *δ* 106.55 (C-2’’), 106.09 (C-2’), 96.68 (C-1), 84.08 (C-5’), 83.89 (C-5’’), 79.71 (C-3’), 79.56 (C-3’’), 77.16 (C-4’’), 76.76 (C-4’), 76.27 (C-3), 73.84 (C-2), 73.00 (C-5), 69.27 (C-4), 65.07 (C-6’), 64.98 (C-6’’), 63.90 (C-1’), 62.52 (C-6), 63.41 (C-1).

ESI-MS: *m/z*: calcd for C_18_H_32_O_16_Na: 527.2, found 527.2 [M+Na]^+^**.**

**β-d-Fructofuranosyl-(2→1)-*β*-d-fructofuranosyl-(2→1)-*β*-d-fructofuranosyl-(2→1)-*α*-d-mannopyranoside (Man-Fru_3_)**

*R_f_* = 0.18 (acetonitrile/water 8:2, 3 ascents); ^1^H NMR (600 MHz, D_2_O): *δ* 5.31 (d, *J=*1.2 Hz, 1H; 1-H), 4.21-4.19 (d, *J=*8.5 Hz, 1H; 3’’’-H), 4.19-4.17 (d, *J=*8.7 Hz, 1H; 3’-H), 4.16-4.14 (d, *J*=8.6 Hz, 1H; 3’’-H), 4.08-4.05 (t, *J=*8.5 Hz, 1H; 4’’-H), 4.06-4.03 (t, *J=*8.5 Hz, 1H; 4’’’-H), 4.03-3.99 (t, *J=*8.5 Hz, 1H; 4’-H), 3.87-3.84 (m, 2H; 2-H, 5-H), 3.84-3.79 (m, 5H; 5’-H, 5’’-H, 1’’’-H_a_, 5’’’-H, 6’’’-H_a_), 3.79-3.75 (m, 5H; 3-H, 6-H_a_, 1’-H_a_, 6’’-H_2_), 3.74-3.69 (m, 6H; 6-H_b_, 1’-H_b_, 6’-H_2_, 1’’-H_a_, 6’’’-H_b_), 3.68-3.62 (m, 2H; 4-H, 1’’-H_b_). ^13^C NMR (150 MHz, D_2_O): *δ* 106.46 (C-2’’), 106.01 (C-2’), 105.88 (C-2’’’), 96.61 (C-1), 84.02 (C-5’), 83.82 (C-5’’), 83.79 (C-5’’’), 80.01 (C-3’’’), 79.58 (C-3’), 79.46 (C-3’’), 77.05 (C-4’’), 76.97 (C-4’’’), 76.61 (C-4’), 76.20 (C-3), 73.77 (C-2), 72.90 (C-5), 69.17 (C-4), 65.00 (C-6’), 64.91 (C-6’’), 64.81 (C-6’’’), 63.86 (C-1’), 63.65 (C-1’’’), 63.42 (C-6), 63.22 (C-1’).

ESI-MS: *m/z*: calcd for C_24_H_42_O_21_Na: 689.2, found 689.2 [M+Na]^+^**.**

**β-d-Fructofuranosyl-α-d-galactopyranoside (Gal-Fru)**

*R*_f_ 0.42 (6:3:1 EtOAc-Isopropanol-H_2_O, 3 ascends); ^1^H NMR (400 MHz, D_2_O): *δ* 5.40-5.39 (d, *J*= 3.9 Hz, 1H, 1-H), 4.18-4.15 (d, *J*= 8.7 Hz, 1H, 3’-H), 4.11-4.07 (dt, *J*= 0.9, 6.4 Hz, 1H, 5-H), 4.04- 4.00 (t, 1H, *J*= 8.7 Hz, 4’-H), 3.99-3.98 (dd, *J*= 0.9, 3.20 Hz, 1H, 4-H), 3.89-3.86 (dd, *J*= 10.5, 3.2 Hz, 1H, 3-H), 3.85-3.76 (m, 3H, 2’-H, 5’-H, 6’-H_2_), 3.70–3.68 (t, *J*= 6.4 Hz, 2H, 6-H_2_),3.64 (s, 2H, 1’-H_2_).^13^C NMR (100 MHz, D_2_O) *δ* 106.2 (C-1’), 94.87 (C-1), 83.86 (C-5’), 79.12 (C-3’), 76.72 (C-4’), 73.99 (C-5), 71.70 (C-3), 71.67 (C-4), 70.55 (C-2), 64.93 (C-6’), 64.08 ( C-1’), 63.44 (C-6).

ESI-MS: *m/z*: calcd for C_12_H_22_O_11_Na: 365.0, found 365.0 [M+Na]^+^**.**

**β-d-fructofuranosyl-(2→1)-*β*- d-fructofuranosyl-(2→1)-*α*- d-galactopyranoside (Gal-Fru_2_)**

*R_f_* = 0.22 (acetonitrile/water 8:2, 3 ascents); ^1^H NMR (600 MHz, D_2_O): *δ* 5.42 (dd, *J=*4.0 1.8 Hz, 1H; 1-H), 4.24-4.22 (dd, *J=*8.8, 2.1 Hz, 1H; 3’-H), 4.15-4.13 (dd, *J=*8.6, 2.1 Hz, 1H; 3’’-H), 4.09-4.08 (“t”, *J*= 5.7 Hz, 1H, 5-H), 4.05-4.02 (dt, *J=*8.4, 2.1 Hz, 1H; 4’’-H), 4.02-3.99 (t, *J=*8.7, 2.1 Hz, 1H; 4’-H), 3.98-3.96 (m, 1H, 4-H), 3.88-3.85 (ddd, *J*= 10.5, 3.3, 2.0, 1H; 3-H), 3.84-3.81 (m, 2H; 5’-H, 5’’-H), 3.80-3.70 (m, 12H; 6-H_2_, 6’-H_2_, 6’’-H_2_), 3.71-3.68 (m, 1H, 6’’-H_b_), 3.72-3.65 (m, 2H, 6’-H_2_), 3.65-3.62 (dd, J= 12.3, 2.1 Hz, 1H, 6’’-H_a_). ^13^C NMR (150 MHz, D_2_O): *δ* 106.47 (C-2’’), 105.90 (C-2’), 95.35 (C-1), 83.56 (C-5’, C-5’’), 79.42 (C-3’), 79.33 (C-3’’), 77.18 (C-4’’), 76.59 (C-4’), 74.19 (C-5), 71.87 (C-4), 71.82 (C-3), 70.78 (C-2), 65.07 (C-6’’), 64.88 (C-6’), 63.68 (C-1’’), 63.59 (C-6), 63.41 (C-1’’).

ESI-MS: *m/z*: calcd for C_18_H_32_O_16_Na: 527.2, found 527.2 [M+Na]^+^**.**

**β-d-fructofuranosyl-(2→1)-*β*-d-fructofuranosyl-(2→1)-*β*-d-fructofuranosyl-(2→1)-*α*-d-galactopyranoside (Gal-Fru_3_)**

*R_f_* = 0.17 (acetonitrile/water 8:2, 3 ascents); ESI-MS: *m/z*: calcd for C_24_H_42_O_21_Na: 689.2, found 689.2 [M+Na]^+^**.**

**β-d-Fructofuranosyl-β-l-fucopyranoside (Fuc-Fru)**

*R*_f_ 0.42 (6:3:1 EtOAc-Isopropanol-H_2_O, 2 ascends); ^1^H NMR (400 MHz, D_2_O) *δ* 4.74-4.71 (d, *J*= 8.0 Hz, 1-H), 4.20- 4.16 (m, 1H, 4’-H), 4.18-4.16 (d, *J*= 7.8 Hz, 1H, 3’-H), 3.87-3.84 (m, 1H, 5’-H), 3.82-3.77 (m, 2H, 6_a_'-H, 5-H), 3.73-3.70 (m, 2H, 6_b_’-H, 4’-H), 3.68-3.65 (d, *J*= 12.6 Hz, 1H, 1’_a_-H), 3.64–3.60 (dd, *J*= 9.9, 3.6 Hz, 1H, 3-H), 3.60-3.57 (d, *J=* 12.6 Hz, 1H, 1’_b_-H), 3.48-3.43 (d, *J*= 8.0, 9.9 Hz, 1H, 2-H), 1.21-1.20 (d, *J*= 6.6 Hz, 3H, 6-H_3_).^13^C NMR (100 MHz, D_2_O) *δ* 106.60 (C-2’), 98.28 (C-1), 84.17 (C-5’), 78.65 (C-3’), 75.21 (C-4’), 75.13 (C-3), 73.79 (C-5), 73.69 (C-4), 72.74 (C-2), 63.51 (C-1’, C-6’), 17.98 (C-6).

ESI-MS: *m/z*: calcd for C_12_H_22_O_10_Na: 349.1, found 349.1 [M+Na]^+^**.**

**β-d-fructofuranosyl-(2→1)-*β*- d-fructofuranosyl-(2→1)-*α*- d-fucopyranoside (Fuc-Fru_2_)**

*R_f_* = 0.39 (acetonitrile/water 8:2, 4 ascents); ^1^H NMR (600 MHz, D_2_O): *δ* 5.28 (d, *J=*4.0 Hz, 1H; 1-H), 4.18 (d, *J=*8.7, 1H; 3’-H), 4.16-4.12 (q, *J*= 6.7 Hz, 1H, H-5), 4.09 (d, *J=*8.5 Hz, 1H; 3’’-H), 3.99 (t, *J=*8.5 Hz, 1H; 4’’-H), 3.95 (t, *J=*8.7 Hz, 1H; 4’-H), 3.84-3.81 (dd,*J*= 3.3, 10.5 Hz, 1H, 3-H), 3.81-3.75 (m, 2H; 5-H, 5’-H), 3.75-3.71 (m, 3H, 1’-H_a_, 6’-H_a_ , 6’’-H_a_), 3.70-3.57 (m, 7H; 1’-H_b_, 6’-H_b_ ,1’’-H_2_, 6’’-H_b_), 1.12 (d, *J*= 6.6 Hz, 3H, CH_3_). ^13^C NMR (150 MHz, D_2_O): *δ* 103.66 (C-2’’), 103.04 (C-2’), 92.68 (C-1), 81.10 (C-5’), 81.06 (C-5’’) 76.61 (C-3’), 76.55 (C-3’’), 74.37 (C-4’’), 73.79 (C-4’), 71.74 (C-4), 69.15 (C-3), 67.71 (C-2), 67.33 (C-5), 62.26 (C-6’’), 61.99 (C-6’), 60.64 (C-1’), 60.33 (C-1’’), 15.35 (CH_3_).

ESI-MS: *m/z*: calcd for C_18_H_32_O_16_Na: 527.2, found 527.2 [M+Na]^+^**.**

**β-d-Fructofuranosyl-(2→1)-*β*-d-fructofuranosyl-(2→1)-*β*-d-fructofuranosyl-(2→1)-*α*-d-mannopyranoside (Fuc-Fru_3_)**

*R_f_* = 0.18 (acetonitrile/water 8:2, 4 ascents); ^1^H NMR (600 MHz, D_2_O): *δ* 5.27 (d, *J=*4.0 Hz, 1H; 1-H), 4.18 (d, *J=*8.7 Hz, 1H; 3’’-H), 4.16-4.13 (q, *J*= 6.8 Hz, 1H, H-5), 4.13 (d, *J=*8.5 Hz, 1H; 3’-H), , 4.09 (d, *J*=8.6 Hz, 1H; 3’’’-H), 4.01 (t, *J=*8.6 Hz, 1H; 4’’’-H), 3.98 (t, *J=*8.5 Hz, 1H; 4’-H), 3.96 (t, *J=*8.7 Hz, 1H; 4’’-H), 3.84-3.81 (dd,*J*= 3.4, 10.5 Hz, 1H, 3-H), 3.80-3.71 (m, 9H; 4-H, 1’-H_a_, 5’-H, 6’-H_a_ ,1’’-H_a_, 5’’-H, 6’’-H_a_, 5’’’-H, 6’’’-H_a_), 3.68-3.57 (m, 7H; 1’-H_b_, 6’-H_b_ ,1’’-H_b_, 6’’-H_b_, 1’’’-H_2_, 6’’’-H_b_)), 1.11 (d, *J*= 6.5 Hz, 3H, CH_3_). ^13^C NMR (150 MHz, D_2_O): *δ* 103.61 (C-2’’’), 103.00 (C-2’’), 102.97 (C-2’), 92.67 (C-1), 81.09 (C-5’’), 81.02 (C-5’’’), 80.99 (C-5’), 77.41 (C-3’), 76.74 (C-3’’), 76.67 (C-3’’’), 74.35 (C-4’), 74.27 (C-4’’’), 73.78 (C-4’’), 71.75 (C-4), 69.15 (C-3), 67.74 (C-2), 67.34 (C-5), 62.19 (C-6’), 62.18 (C-6’’’), 61.99 (C-6’’), 60.80 (C-1’, C-1’’), 60.30 (C-1’’’), 15.34 (CH_3_).

ESI-MS: *m/z*: calcd for C_24_H_42_O_21_Na: 689.2, found 689.2 [M+Na]^+^**.**

**β-d-Fructofuranosyl-α-d-xylopyranoside (Xyl-Fru)**

*R*_f_ 0.46 (6:3:1 EtOAc-Isopropanol-H_2_O, 2 ascends); ^1^H NMR (400 MHz, D_2_O) *δ* 5.30-5.29 (d, *J*= 3.6 Hz, 1H, 1-H), 4.17-4.15 (d, *J*= 8.9 Hz, 1H, 3’-H), 4.07-4.02 (t, *J=* 8.9 Hz, 1H, 4’-H), 3.85-3.81 (dt, *J*= 8.9, 2.8 Hz, 1H, 5’-H), 3.78-3.74 (2d, *J*= 2.8 Hz, 2H, 6_a_’-H, 6_b_’-H), 3.68-3.60 (m, 2H, 3-H, 5-H), 3.60 (s, 2H, 1’-H_2_), 3.56-3.54 (m, 1H, 4-H), 3.50-3.46 (dd, *J*= 9.9, 3.6 Hz, 1H, 2-H). ^13^C NMR (100 MHz, D_2_O) *δ* 106.35 (C-2’), 94.97 (C-1), 84.01 (C-5’), 78.83 (C-3’), 76.26 (C-4’), 75.40 (C-3), 73.67 (C-2), 71.82 (C-4), 64.45 (C-6’), 64.38 (C-5), 63.48 (C-1’).

ESI-MS: *m/z*: calcd for C_11_H_20_O_10_Na: 335.0, found 335.0 [M+Na]^+^**.**

**β-d-Fructofuranosyl-(2→1)-*β*- d-fructofuranosyl-(2→1)-*α*-d-xylopyranoside (Xyl-Fru_2_)**

*R_f_* = 0.29 (acetonitrile/water 8:2, 3 ascents); ^1^H NMR (400 MHz, D_2_O): *δ* 5.33 (d, *J=*3.9 Hz, 1H; 1-H), 4.23-4.21 (d, *J=*8.8 Hz, 1H; 3’-H), 4.14-4.12 (d, *J=*8.5 Hz, 1H; 3’’-H), 4.07-4.02 (t, *J=*8.6 Hz, 1H; 4’-H), 4.04-4.00 (t, *J=*8.2 Hz, 1H; 4’’-H), 3.86-3.78 (m, 2H, 5’-H, 5’’-H), 3.79-3.69 (m, 4H, 6’’-H_2_, 1’-H_a_, 6’-H_a_), 3.68-3.59 (m, H, 1’-H_a_, 6’-H_a_ 1’’-H_2_, 5-H2, 3-H, 4-H), 3.50-3.46 (dd, J= 9.7, 3.9 Hz, 1H, 2-H). ^13^C NMR (100 MHz, D_2_O): *δ* 106.12 (C-2’, C-2’’), 95.47 (C-1), 84.11 (C-5’), 83.94 (C-5’’), 79.43 (C-3’, C-3’’), 77.25 (C-4’’), 76.39 (C-4’), 75.65 (C-3), 73.98 (C-2), 72.05 (C-4), 65.15 (C-6’’), 64.69 (C-5), 64.55 (C-6’), 63.47 (C-1’), 63.21 (C-1’’).

ESI-MS: *m/z*: calcd for C_17_H_30_O_15_Na: 497.2, found 497.2 [M+Na]^+^**.**

**β-d-Fructofuranosyl-(2→1)-*β*-d-fructofuranosyl-(2→1)-*β*-d-fructofuranosyl-(2→1)-*α*-d-xylopyranoside (Xyl-Fru_3_)**

*R_f_* = 0.22 (acetonitrile/water 8:2, 3 ascents); ESI-MS: *m/z*: calcd for C_23_H_40_O_20_Na: 659.2, found 659.2 [M+Na]^+^**.**

**Supporting Figure 1**

**Lipopolysaccharide interactions with cell surface-located Toll-like receptors 2 and 4**

The different shapes of bacterial lipopolysaccharides (LPS) are keys for the identification of their target receptor (simplified adaption from [17]). Here, the shape of LPS ligands is determined by the grade of fatty acid substitution. The distinct receptor binding mode depending on the molecular conformation is supposed to be mimicked by the oligosaccharides tested in this study.


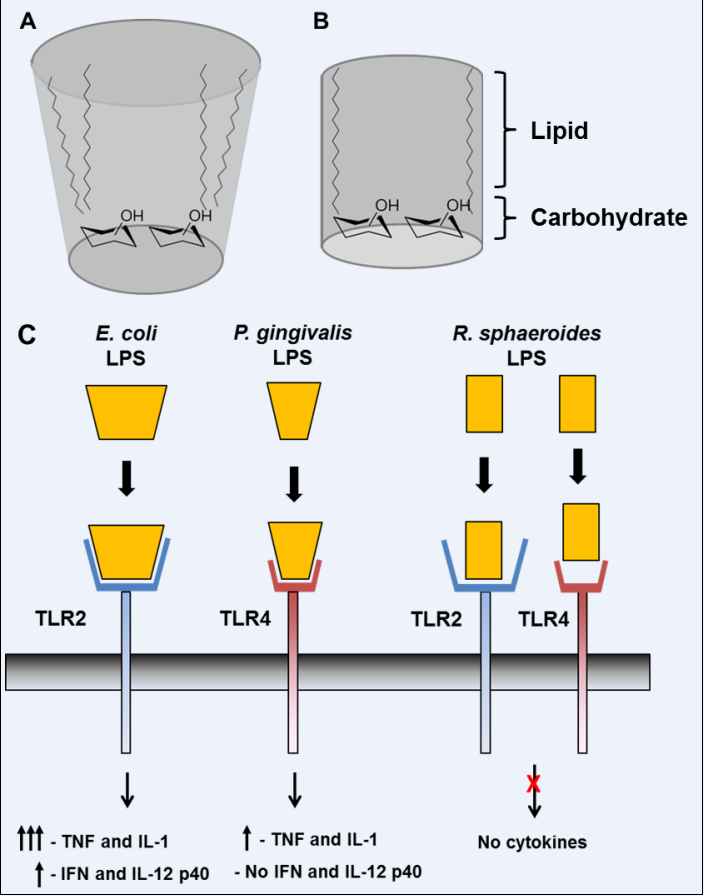

Supplement: Additional file 1: Figure S1 — Lipopolysaccharide interactions with cell surface-located Toll-like receptors 2 and 4. The different shapes of bacterial lipopolysaccharides (LPS) are keys for the identification of their target receptor (simplified adaption from [17]). Here, the shape of LPS ligands is determined by the grade of fatty acid substitution. The distinct receptor binding mode depending on the molecular conformation is supposed to be mimicked by the oligosaccharides tested in this study. [file 1472-6750-12-90-S1.docx]
